# Heterogeneity of Neuroinflammatory Responses in Amyotrophic Lateral Sclerosis: A Challenge or an Opportunity?

**DOI:** 10.3390/ijms21217923

**Published:** 2020-10-25

**Authors:** Giada Cipollina, Arash Davari Serej, Gianluca Di Nolfi, Andrea Gazzano, Andrea Marsala, Mauro G. Spatafora, Marco Peviani

**Affiliations:** Department of Biology and Biotechnology “L. Spallanzani”, University of Pavia, via Ferrata 9, 27100 Pavia, Italy; giada.cipollina@unipv.it (G.C.); arash.davariserej@unipv.it (A.D.S.); gianluca.dinolfi01@universitadipavia.it (G.D.N.); andrea.gazzano01@universitadipavia.it (A.G.); andrea.marsala01@universitadipavia.it (A.M.); maurogiuseppe.spatafora01@universitadipavia.it (M.G.S.)

**Keywords:** neuroinflammation, microglia, single-cell RNAseq, spatial transcriptomics, PET

## Abstract

Amyotrophic Lateral Sclerosis (ALS) is a complex pathology: (i) the neurodegeneration is chronic and progressive; it starts focally in specific central nervous system (CNS) areas and spreads to different districts; (ii) multiple cell types further than motor neurons (i.e., glial/immune system cells) are actively involved in the disease; (iii) both neurosupportive and neurotoxic neuroinflammatory responses were identified. Microglia cells (a key player of neuroinflammation in the CNS) attracted great interest as potential target cell population that could be modulated to counteract disease progression, at least in preclinical ALS models. However, the heterogeneous/multifaceted microglia cell responses occurring in different CNS districts during the disease represent a hurdle for clinical translation of single-drug therapies. To address this issue, over the past ten years, several studies attempted to dissect the complexity of microglia responses in ALS. In this review, we shall summarize these results highlighting how the heterogeneous signature displayed by ALS microglia reflects not only the extent of neuronal demise in different regions of the CNS, but also variable engagement in the attempts to cope with the neuronal damage. We shall discuss novel avenues opened by the advent of single-cell and spatial transcriptomics technologies, underlining the potential for discovery of novel therapeutic targets, as well as more specific diagnostic/prognostic not-invasive markers of neuroinflammation.

## 1. Introduction

Amyotrophic Lateral Sclerosis (ALS) is a fatal chronic neurodegenerative disease leading to a progressive loss of cortical (upper motor neurons, UMN), brainstem and spinal cord (lower motor neurons, LMN) motor neurons. Despite a high number of potential drugs validated at the preclinical level and tested in clinical trials in the last 20 years, ALS is still an incurable disease [1]. Among the several therapeutic approaches tested up to now, many were based on chronic administration of neuroprotective factors (e.g., trophic or anti-apoptotic factors, treatment with anti-glutamatergic drugs or compounds enhancing proteasome or mitochondrial metabolic activity) or anti-inflammatory molecules (such as cyclooxygenase inhibitors or minocycline). These strategies suffered of limited efficacy. Reasons for such failure could be the induction of pharmacological tolerance during the chronic treatment or the lack of specificity for selected cell types (glia versus neurons). In fact, despite that neurons are the primary cell type affected in ALS, accumulating evidence suggest that neuroinflammatory responses actively participate in the pathology and have an impact on the neurodegeneration.

Neuroinflammation is defined as reactive astrogliosis, microgliosis and lymphocytes infiltration in the central nervous system (CNS). It has been recognized as a distinctive marker of pathology that goes along with neuronal demise not only in ALS, but also in several other neurodegenerative disorders, ranging from acute brain/spinal cord injury to Alzheimer’s disease (AD), frontotemporal dementia (FTD), or Parkinson’s disease (PD) [2,3,4]. One of the major drivers of reactive glial responses is the accumulation of proteinaceous aggregates both at the intracellular level (leading to neuronal demise and the release of pro-inflammatory cytokines) as well as in the extracellular space [5]. This represents a neuropathological hallmark of ALS, as well as other neurodegenerative diseases, e.g., several types of inclusions have been reported in neuronal perikaryon and neurites, such as phosphorylated neurofilaments in ALS, ubiquitinated skein-like and Lewy-body like inclusions in ALS and PD, transactive response DNA binding protein 43KDa (TDP-43) in ALS and FTD; tau-positive neurofibrillary tangles in AD. On the other hand, extracellular aggregates include β-amyloid plaques in AD and misfolded superoxide dismutase 1 (SOD1) in ALS [6]. Additionally, glial cells participate in an intricate signaling network with neurons, characterized by the release of cytokines and neurotransmitters, trophic factors, and exosomes [7,8]. Overall, this complex signaling shapes the neuronal microenvironment, leading to a neurosupportive or alternatively to a pro-degenerative milieu depending on disease stage and the phenotype of the activated glial cells. Understanding the mechanisms regulating the switch from a neurosupportive to a neurotoxic phenotype is critical to develop more specific and efficacious therapies. As an example, systemic administration of minocycline (an anti-inflammatory drug) in a mouse model of ALS resulted in neuroprotective effects by dampening microgliosis when treatment started before symptom onset, while it worsened the pathology by inducing a strong astrocytosis and neurotoxic microglia phenotype when the treatment was performed during the symptomatic stage of the disease [9,10]. This further supports the need for a deeper understanding of the changes occurring in glial cell phenotype at different stages of the disease and in different CNS districts characterized by variable extent of neuronal demise.

In the past years, several studies performed on ALS animal models and patients focused the attention on the role played by microglia in the pathology [11,12]. A very complex picture was depicted, characterized by an intrinsic heterogeneity of glial cells responses, which reflects CNS region-specific differences in the extent of neuronal demise, in the capability to respond to damage and, potentially, in the susceptibility to pharmacological treatments.

**Figure 1:**
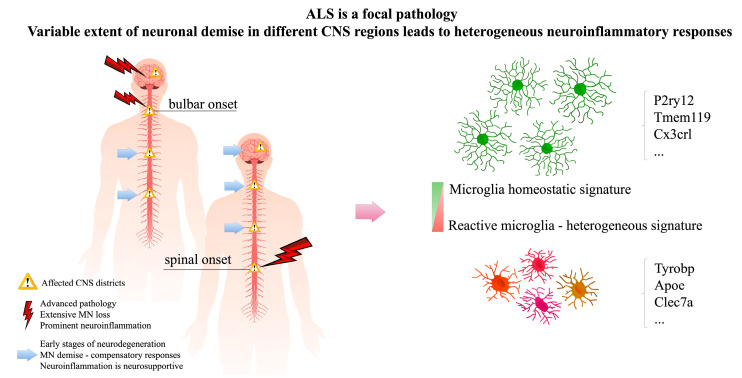
Amyotrophic Lateral Sclerosis (ALS) is a complex pathology. There is variability in the extent of neuronal demise in different central nervous system (CNS) districts affected by the disease. This leads to heterogeneity of neuroinflammatory (microglia) responses, characterized by variable engagement in the attempt to cope with neuronal demise.

**Figure 2:**
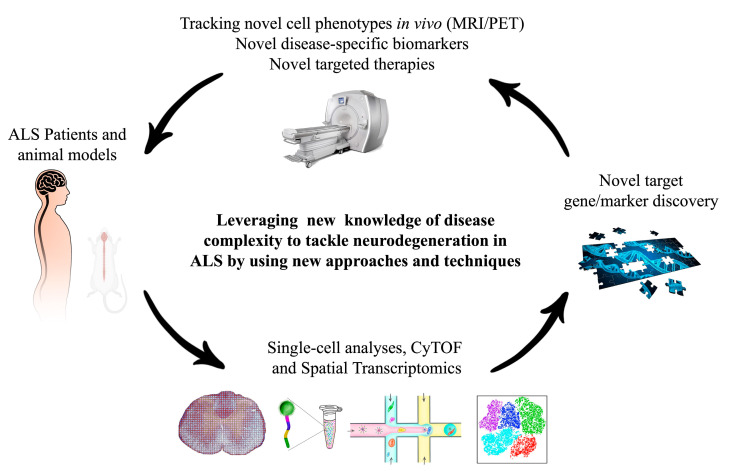
Strategies to investigate the complex microglia signature in ALS include single-cell and spatial transcriptomics approaches or multi-omics techniques such as cytofluorometry by time-of-flight (CyTOF). These will allow to identify novel targets that could be exploited therapeutically or for magnetic resonance imaging (MRI)/positron emission tomography (PET) imaging studies.

In this review, we shall present an update on the multifaceted functions exerted by microglia in ALS (Figure 1), highlighting how novel single-cell approaches could help to navigate through the heterogeneous phenotypes manifested by these cells in the CNS during the disease, paving the way for potential identification of novel therapeutic targets and, possibly, improved diagnostic/prognostic markers (Figure 2).

## 2. ALS Is a Focal Pathology, Neuroinflammatory Responses Are Intrinsically Heterogeneous

The most common initial presentation of ALS is focal asymmetric distal weakness accompanied by muscular atrophy, which reflects the presence of specific foci of neuronal dysfunction in restricted CNS areas. In fact, ALS probably begins a long time before its clinical manifestations, since a substantial number of motor neurons can be lost before any clinical signs develop. Like in any biological system, the organism initially senses the early neuronal dysfunctions and tries to compensate through re-innervation from nearby motor neurons. This allows to preserve the motor function, until more than 50% of motor units are lost; at this point the clinical symptoms appear and the pathology progresses rapidly [13,14].

Clinical manifestations of the classic forms of ALS present a combination of UMN and LMN signs, including fasciculations, muscle weakness and atrophy (LMN signs) or hyperreflexia and slowness of movements (UMN signs). However, increasing evidence highlight heterogeneous disease manifestations from patient to patient, characterized by predominant involvement of different motor districts and variable extent of extra-motor deficits or involvement of frontotemporal degeneration. This leads to a high variety of phenotypic manifestations leading to different disease trajectories. The heterogeneity of disease manifestations influences not only the diagnosis, that could be complicated in the early stages of the pathology due to the great variability in the extent and localization of motor system involvement in different patients [15], but it could also affect the responsiveness to treatment. In fact, patients with poor prognosis might not benefit from one treatment in the same manner as patients with a slower disease progression. This explains the quest for imaging [16,17,18] or biofluid [19] biomarkers that could help to stratify patients according to the prevalent disease manifestations and to prognosis predictors.

The neuroinflammatory responses fit perfectly in this complex picture: several evidences highlight that activation of astrocytes and microglia exerts a neurosupportive function in the early stages of neuronal demise, contributing to support the compensatory responses. However, over time, the chronic neurodegeneration becomes overwhelming and glial cells shift towards a more neurotoxic phenotype eventually contributing to the spreading of the disease to other CNS districts and to the progression of the pathology towards the late stages [12,20,21,22,23]. As evidenced by several neuropathological studies [24,25,26], microgliosis and astrocytosis are hallmarks that appear very early in the disease, consistent with the emergence of the first signs of neuronal demise and long before the occurrence of massive neurodegeneration that leads to the onset of symptoms. Thus, a scenario can be envisaged where, in the patient at the early stages of the disease, some CNS districts might be already severely affected (with profound neuronal loss and ongoing neurotoxic neuroinflammatory responses) whereas in other CNS regions, where functionality is still preserved, compensatory mechanisms (i.e., collateral re-innervation and neurosupportive gliosis) could still be in place. A treatment targeting at the same time and in the same manner both degenerated and partially preserved CNS regions could result in a detrimental outcome, e.g., by inhibiting endogenous compensatory neuroprotective responses in one region while trying to counteract pro-degenerative pathways in another already compromised area.

As an example of the complex consequences of this phenomenon, we present the challenges of modulating nuclear factor kappa-light-chain-enhancer of activated b cells (NF-κB) (a transcription factor that acts as a master regulator of neuroinflammation) as a therapeutic target for ALS. NF-κB activation in microglia cells in SOD1.G93A mice is a potent driver of the conversion to a cytotoxic phenotype, contributing to MNs death in this model. In fact, genetic inhibition of this pathway (via crossbreeding of SOD1.G93A with mice carrying a microglia-specific knock-out for Inhibitory-κB Kinase (IKK) leading to constitutive inhibition of NF-κB pathway in these cells) was neuroprotective [27]. However, the role of NF-κB in different glial cell types is still controversial: NF-κB resulted in the highest-ranked regulator of inflammation in gene array data-sets from astrocytes derived from human post mortem ALS patients [28]; NF-κB was found upregulated in glia and neuronal cells from familial and sporadic ALS patients with TDP-43 (one of the hallmarks of ALS) acting as a co-activator of the p65 subunit of the NF-κB complex [29]. Recent evidence highlighted a disease stage-dependent activation of the inflammatory pathway NF-κB in astrocytes; this determines an enhancement of neurosupportive microglia early in the pathology, followed by a shift towards induction of a pro-inflammatory phenotype later [30,31]. Endo and colleagues also demonstrated that overproduction of transforming growth factor beta (TGF-β) by astrocytes in ALS may contribute to inhibit the neuroprotective functions of microglia, by decreasing the expression of insulin growth factor 1 ( IGF-1) [23]. Despite being therapeutically relevant, targeting NF-κB is complicated due to its diverse functions in different cell types including neurons. In fact, neuronal NF-κB activation is involved in learning and memory, by influencing dendritic arborization and axonal outgrowth [32,33]; unspecific downregulation of neuronal NF-κB can indeed exacerbate neurodegeneration in some disease models [34,35]. Thus, a deeper understanding of the phenotype displayed by glial cells in different regions of the CNS during disease progression is necessary. This would help to define more efficacious therapeutic approaches that should be designed to engage specific pathological pathways in a precise time-window of the disease.

## 3. The Signature of Activated Microglia in ALS Is Disease Specific

Microglial cells belong to the monocyte/macrophage cell lineage; they originate from the yolk sac and colonize the CNS early during development. Mature microglial cells in adult nervous system are believed to serve as the innate immune defense for brain and spinal cord, by providing constant surveillance versus viral, bacterial or fungal infections. Microglia can actively participate in CNS remodeling in healthy conditions through interaction with astrocytes and by sensing the functionality of neuronal synapses (aiding synaptic pruning), or by removing the degenerating neurons through phagocytosis. In response to an insult, microglial cells undergo a rapid functional activation, as evidenced by modification of the cell morphology, antigen presentation, surface receptor expression, as well as by production and release of reactive oxygen species (ROS), cytokines and growth factors. The multifaceted functions of microglia in health and disease have been traditionally depicted based on morphological and histological evidence, leading to the definition of resting microglia (present in the healthy brain) as opposed to activated microglia (found in pathological conditions). Resting microglia is CD45^low^CD11b^+^Iba1^+^CXC3R1^+^CD68^-^, it is characterized by small cell body and large and highly branched arborization of the cytoplasm; it constantly scans the environment participating in immune surveillance and synaptic pruning. Activated microglia is CD45^high^CD11b^+^Iba1^+^CXCR3^+^CD68^+^, it is induced by pro-inflammatory cytokines or other stimulatory signals and/or neurotransmitters released by neighboring cells and it can display different morphologies ranging from small cell body and thick arborization to amoeboid-like shape (no arborization and large cell body), typical of highly macrophagic cells, depending on the type of challenging stimulus and the microenvironment [36].

Microglia cells in ALS are involved in spreading of the pathology to other CNS districts and in progression of the disease towards late stages [20,21,22]. Notably, microglia-restricted deletion of the mutant SOD1 gene did not affect symptom onset, but radically slowed disease progression [37]. Similarly, bone marrow transplantation of wild-type microglia into SOD1.G93A; PU.1^-/-^ mice (which lack CNS microglia at birth) prolonged the disease duration and overall survival [38]. Thanks to the application of RNA-seq studies, conducted on microglia cells directly isolated from mutant SOD1 mice and through analyses on post-mortem spinal cord samples from familial (fALS) and sporadic (sALS) patients, it has been possible to depict microglia phenotypes based on their gene-expression signatures, rather than just on morphological features. These studies highlighted a loss of microglia homeostatic signature (Table 1a) and acquisition of a neurodegeneration-specific phenotype in ALS (Table 1b) [39]. Interestingly, the pathological signature of ALS microglia was unique, in the sense that it was not shared with the signature of lipopolysaccharide (LPS)-stimulated macrophages (Table 1c) or other cell types. Importantly, this neurodegeneration-specific phenotype was characterized by concurrent up-regulation of neurotrophic genes (such as *IGF-1*, *DAP12* and *progranulin*) and pro-oxidative/cytotoxic genes, such as NADPH-oxidase (*NOX2*), optineurin (*OPTN*) and matrix metallopeptidase 12 (*MMP-12*) (Table 1b). Endo and colleagues demonstrated that overproduction of TGF-β by astrocytes in ALS may contribute to inhibit the neuroprotective functions of microglia, by decreasing the expression of IGF-1 [23]. On the other hand, increased ROS production by microglia may contribute to increased oxidation (i.e., inactivation) of motor neuronal IGF-1 receptors [40], thus, affecting the possibility to induce a neuroprotective response in these cells. Microglia also release exosomes [41,42], thus, increasing interleukin 1 beta (IL-1β) within the extracellular environment and propagating inflammation [43]. Altogether, these observations suggest that an endogenous, initial pro-regenerative response of microglia is overcome by stressful stimuli (including oxidative stress) accumulating over time, leading to changes in the microenvironment with detrimental outcome for motor neurons. Therefore, microglia should be considered as a key target in ALS therapeutic attempts with the goal of fostering its neuroprotective and pro-regenerative activity. However, how pro-degenerative and neurosupportive pathways are regulated in activated microglia in ALS remains controversial.

The discovery of miR-155 as one of the pro-inflammatory miRNAs most highly upregulated in the spinal cord resident microglia in SOD1 mice and in post-mortem samples from ALS patients helped to identify one possible regulator of the complex phenotype displayed by microglia in ALS [44,45]. Upregulation of miR-155 was found associated with a loss of the molecular signature that characterizes homeostatic microglia in favor of a more neurotoxic phenotype [45], suggesting that miR-155 may represent a crucial target acting as a master-regulator for restoration of a physiological neurosupportive microglia function. In line with this observation, treatment with a miR-155 inhibitor partially restored homeostatic microglia genes and prolonged survival of SOD1 mice [45,46].

Altogether these data confirm the importance of modulating microglia reactivity to modify disease progression. However, since both neuroprotective and cytotoxic functions have been identified in reactive microglia cells during disease progression [23,39], it remains to be clarified whether these multifaceted functions belong to one single and complex phenotype displayed by the same population of activated cells, or rather the concurrent upregulation of pro-inflammatory and neurosupportive genes results from the coexistence of different cell phenotypes (either pro-inflammatory or neurosupportive) in the same CNS region analyzed. In fact, given that standard RNA-seq approaches, such as the ones described above, started from tissue homogenates, the results represent a snapshot of the mRNA expressed by the pool of cells that were retrieved from the tissue at time of analysis; in other words, single cell identity is not preserved in these studies.

## 4. Single-Cell and Spatial Transcriptomics Approaches: Powerful Tools to Unveil the Complexity of Neuroinflammatory Responses

Recent development of microfluidic droplet-based technologies, allowing high throughput single-cell analysis, opened the way for thorough investigation of cellular heterogeneity in different tissues including the brain in health and disease, with seminal discoveries in neuronal and microglia physiology [62]. These technologies could offer valuable insights into the complex and intricate pathways regulating neuron/glia interactions in ALS and provide tools to identify: (i) critical factors to improve available single-drug therapies; (ii) novel candidate molecular targets for innovative therapeutic approaches; (iii) novel cell-specific markers to be exploited for targeted drug/gene delivery or to track (by magnetic resonance imaging (MRI)/positron emission tomography (PET)) the presence and distribution of specific cell phenotypes critical for the pathology or susceptibility to treatment.

A seminal paper from Keren-Shaul et al. applied single-cell RNAseq on the 5xFAD transgenic Alzheimer’s disease mouse model [48,63]. The authors uncovered the existence of a subset of microglia cells, which they defined disease-associated microglia (DAM), which was uniquely present in Alzheimer’s disease (AD) but not in wild type animals. The signature of DAM microglia is characterized by a reduction of the expression levels of microglia homeostatic genes (i.e., *P2ry12/P2ry13*, *Cx3cr1*, and *Tmem119*) and upregulation of many genes including known risk factors for AD (i.e., *Apoe, Ctsd, Tyrobp, Lpl,* and *Trem2*) (see reference [56] for full list of DAM genes). CD9, Itgax (CD11c), Clec7a, and CD63 were identified as potential DAM markers and used to enrich DAM microglia from 5xFAD brains, allowing to study the proportion of this cell subtype along disease progression and its tissue distribution. According to this analysis, microglia displays a transition from homeostatic towards DAM signature as a function of disease progression, i.e., downregulation of *P2ry12/13* and *Cx3cr1* and upregulation of *Tyrobp* and *Apoe* characterizes the early stages of the disease whereas in the advanced stages upregulation of *Cst7, Lpl*, and *Trem2* is reported [56]. Interestingly, there is regional specificity for the DAM signature in 5xFAD mice (DAM microglia is found in the cortex, affected by the disease, whereas it is absent from the cerebellum, not involved in the neurodegeneration) [56]. Moreover, microglia cells surrounding Aβ plaques stain positive for DAM markers [56], suggesting an involvement of DAM microglia with scavenging of extracellular aggregates and cellular debris. This is consistent with the results of gene set enrichment analysis of DAM-specific genes, highlighting upregulation of lysosomal/phagocytic pathways, endocytosis and immune responses [56].

One important finding of Keren-Shaul and colleagues is that DAM microglia appears to be a cell population not associated exclusively with AD; in fact, the authors identify the DAM signature also in microglia isolated from the spinal cord of SOD1.G93A mouse model of ALS both in the early and in the late stages of the disease (Table 1c) [56]. Interestingly, a signature consistent with DAM (22 genes out of the 77 identified as DAM markers in humans) has been recently highlighted in the motor cortex of ALS patients [56]. Notably, the motor cortex is one of the CNS regions that is more vulnerable and early affected in ALS patients; several evidence highlight an exacerbation of innate immune responses with microgliosis in ALS motor cortex strengthening the correlation between motor neuronal demise and neuroinflammation in this CNS region. This suggests that DAM could be a general genetic program that is activated to aid the clearance of misfolded and aggregated proteins that accumulate in age-related neurodegenerative diseases such as AD and ALS. However, it still needs to be clarified whether DAM is part of a neuroprotective glial response that is activated late during ALS pathology to try to mitigate the disease, or rather it is an over-reactive response to neuronal demise that eventually contributes to amplify the damage in the affected CNS regions. Interestingly, *Trem2* (one of the major drivers of DAM signature) exacerbates AD manifestations when it is absent from microglia at the late stage of the disease, but not at the early stages [64]. Moreover, genetic variants of Triggering Receptor Expressed on Myeloid Cells 2 (*Trem2* have been associated with increased risk of AD, ALS, and PD [65]. However, how alterations of *Trem2* directly affect ALS pathology has not been addressed in preclinical models, yet. Thus, it cannot be predicted whether the functions of DAM microglia hypothesized for AD could apply also to ALS. For instance, DAM signature has been uncovered also in a subpopulation of microglia cells (defined as proliferative region-associated microglia, PAM) in healthy mice [66]. This cell type appears transiently only in early stage of mouse brain development (post-natal day 7), its DAM-like signature polarization occurs in a *Trem2*-*Apoe* independent manner (differently from what was found in AD mouse models) and it seems to be involved in the regulation of myelination during development. Additionally, an inverse correlation was found between the proportion of microglial cells and the amount of phosphor-TDP43 aggregates in the cortex of affected ALS patients [51]. This and other preclinical in vivo and in vitro evidence suggest that mutant TDP43 might play a role also in the modulation of neuroinflammatory responses in ALS, by hindering or inhibiting the phagocytic responses of reactive microglia precipitating the pathology [64]. Overall, these data further strengthen the need for a more comprehensive understanding of the molecular mechanisms regulating the heterogeneous glial cell responses reported at different stages of the disease and the switch from neurosupportive to pathologic microglia in ALS. Many RNAseq studies (performed on whole tissue or at single-cell level) focused the observations only on whole spinal cord or whole brain or just the brainstem. Extending the analyses to cover simultaneously multiple CNS regions variably affected by the pathology at different stages of the disease could shed light on the mechanisms regulating the complex microglia cell responses in ALS. This is supported by the recent observation of regional heterogeneity of microglia phenotypes in human brain (highlighted by multiplexed single-cell mass spectrometry) [67,68].

Recent years heralded great advances in the capability to dissect the genetic signatures of cells within the tissue in situ, through the advent of spatial transcriptomics (ST). This is a high throughput technology used to generate complete transcriptomics data while maintaining the information of their spatial distribution on a tissue slice [69]. ST exploits custom-made tissue slides containing spotted arrays of specialized mRNA-capturing probes that bind and capture the mRNA from the tissue slice. The positional information for each spot is maintained by using a unique spatial barcode for each probe. Slide-seq (an alternative ST method) was developed more recently by using barcoded oligos attached on beads immobilized on the glass slide, enabling transcriptome-wide measurements with 10-micron spatial resolution [70]. This technique has been recently further improved (now called Slide-seqV2) allowing to achieve better mRNA capturing sensitivity and near-cellular resolution [71].

Maniatis et al. used ST to investigate the spatial distribution of disease-driven gene expression profiles in ALS mouse model at different stages of the disease and in post-mortem spinal cord samples from human ALS patients [72]. Also, by combining single-cell RNAseq and ST the authors aimed at identifying the master-regulator genes involved in the switch and maintenance of a particular disease-associated gene expression signature or cellular phenotype. This study highlighted alterations in several cell types (ranging from motor neurons to micro- and macro-glial cells), reporting for instance a significant change in the expression of some ALS-related genes such as Matr3, Kif5a, and Pfn1. However, for the sake of this review, we shall comment only the results obtained on microglia cells. In fact, thanks to ST approach, the authors demonstrate early dysfunction of microglia before symptom onset highlighting, for the first time, regional differences between the gene signatures displayed in the white versus the gray matter. More importantly, their observations both in the ALS mouse model and on patients’ samples support the correlation between the anatomical proximity to the site of symptom onset and the severity of ALS pathology (evidenced by neurodegeneration-specific gene signatures). For instance, the gene-expression module 1 which includes genes involved in Vascular Endothelial Growth Factor (VEGF) and glutamatergic signaling is attenuated in lumbar sections from patients that have a lower limb site of onset. Furthermore, human expression module 3 (containing genes enriched in several pathways including sphingolipid biosynthesis, endocannabinoid system, and Wingless/Integrated (WNT) signaling) is attenuated across spinal cord sections at sites proximal to symptoms onset and is most noticeable in the posterior white matter and anterior horns. The gene-expression program that includes the homeostatic microglia gene Sall1 is increased in the white matter of control and presymptomatic ALS animals, whereas it is attenuated in the white matter of end-stage ALS animals (the attenuation meaning a loss of homeostatic function and induction of a phagocytic pro-inflammatory phenotype). Among the genes identified in the DAM signature, *Tyrobp* (also named *Dap12*) expression is up-regulated in the ventral horn spinal cord and ventral white matter already at the presymptomatic stage and before *Trem2* induction. *Lpl* and *B2m* (two DAM-associated genes) are simultaneously up-regulated (presymptomatically) specifically in the ventral horn, whereas *Apoe* (DAM-associated) and *Cx3cr1* (homeostatic gene) are not. Expression of these latter genes becomes widely up-regulated in spinal cords of symptomatic mice [58]. Notably, the *Trem2*/DAP12 signaling was identified as the principal regulator that switches microglia from a homeostatic to a DAM state. The heterogeneous microglia signatures highlighted through ST (especially in the white matter already at presymptomatic stage) further supports the existence of region-specific clues (cytokines or other factors released locally by dying motor neurons or other glial cells) that drive the switch towards a neurosupportive or rather a cytotoxic phenotype.

Besides the mentioned advantages of ST, it should be recognized that despite great improvements in the spatial resolution, all information between mRNA capture-spots is lost. Moreover, the ST gene expression map is generated by combining the info on gene expression obtained from all kinds of cells present in the tissue, which might create noise. On the other hand, single-cell RNA-seq provides also the possibility of employing even further experiments ahead, such as flow cytometry by time-of-flight mass spectrometry (CyTOF) to sort cells with specific markers of interest and provide the possibility of high-dimensional analysis of cell surface markers, signaling molecules and cytokines on the brain cells at the single-cell level [58]. Indeed, we should most likely target a combination of these techniques to achieve a deeper understanding of the complex signaling network that regulates the phenotypes displayed by microglia and other cell types in different anatomical regions and disease stages.

## 5. Neuroinflammation as a Diagnostic/Prognostic Marker

Since the first description of ALS in 1869, by the French neurologist J. M. Charcot, the distinct “myelin pallor” and hardness of the lateral columns of spinal cord at autopsy, due to the loss of axons and massive micro- and macro-gliosis in the corticospinal tract, have been recognized as a specific feature of the pathology. Subsequent studies expanded the former Charcot’s observation, highlighting reactive microgliosis as hallmark of the pathology also in the motor cortex, motor nuclei of the brainstem, spinal cord ventral horns, and within the cerebrospinal fluid (CSF) of ALS patients [73]. These findings have attracted great efforts for addressing neuroinflammation as a diagnostic/prognostic factor and a possible target for therapy. Molecular imaging techniques, ranging from magnetic resonance imaging (MRI) to positron emission tomography (PET), have become powerful tools to evaluate neuroinflammation in the pre-clinical and clinical settings, since they offer the advantage of visualizing the markers of inflammation non-invasively, by means of specific probes. Given the close correlation between the inflammatory process and neuronal sufferance, this holds the potential for sensitive and early detection of areas of neuronal demise, as well as for monitoring the disease progression and the response to therapeutic interventions. Non-invasive assessment of microglial activation in ALS patients can be performed through neuroimaging of the 18 kDa translocator protein (TSPO) using selective TSPO radioligands [74,75,76]. TSPO, formerly named peripheral benzodiazepine receptor, is a ubiquitous high-affinity cholesterol transporter found primarily in steroid-synthesizing cells [77]. It is a transmembrane protein localized in the outer mitochondrial membrane, where it integrates hormone- and redox-sensitive cellular response pathways by: (i) regulating mitochondria cholesterol uptake [78]; (ii) interacting with the voltage-dependent anion channel (VDAC) [78]; and (iii) modulating the activity of the membrane permeability transition pore (MPTP) [79], with impact on cellular survival and proliferation. To a lesser extent, TSPO binding sites have been also reported in the endoplasmic reticulum (ER), cell nucleus [80] and plasma membrane [81], probably due to the formation of membrane contact sites in regions where different organelles become in close juxtaposition with mitochondria [82], the so called mitochondria-associated ER membranes (MAMs) [83]. This suggests that TSPO may work as a wider mediator of cellular metabolic adaptation to various extra- and intra-cellular stimuli. In imaging studies, TSPO is hardly detectable in a healthy brain, whereas it becomes highly upregulated under pathological conditions, such as multiple sclerosis or stroke [84] and in chronic neurodegenerative diseases, such as Alzheimer’s, ALS [85], or some lysosomal storage disorders [86,87,88]. Microglia cells are the main cell type responsible for increased TSPO signal in ALS. Some reports also point to a contribution of reactive astrocytes to the increased brain TSPO signal density [89,90]. However, post-mortem human studies have suggested that increased binding of TSPO ligands overlaps more prominently with CD68^+^ activated microglia than with astrocytes, at least in the motor cortex [91]. Therefore imaging of TSPO has been proposed as a useful and sensitive marker to monitor microglia-related neuroinflammation in the brain [92]. Historically, [^11^C]-PK11195 (a 3-isoquinolinecarboxamide) is the first TSPO radiotracer extensively used for PET imaging studies in several neurodegenerative diseases including ALS [93], where significantly increased binding was reported in motor cortex, pons, dorsolateral prefrontal cortex and thalamus. In particular, the PK11195 signal in the motor cortex of ALS patients correlated with the burden of upper motor neuron demise [77,94,95]. However, some limitations were pointed out [77]: a poor signal-to-noise ratio, high lipophilicity and a short half-life of the radioligand of only 20 min, which restricts the use of PK11195 to places where a cyclotron is present on site. For this reason, many alternative TSPO ligands have been explored. [^18^F]DPA-714 and [^11^C]-PBR28 are second-generation ligands that showed promising results in many animal models and in patients [96,97]. [^18^F]DPA-714, tested in a direct comparison with [^11^C]PK11195 in a rat model of cerebral ischemia, provided a higher signal-to-noise ratio and better specificity for the pathological CNS districts (with little-no uptake in the contralateral healthy hemisphere) as compared to PK11195. [18F]DPA-714 binding was increased in the motor cortex in a cohort of ALS patients with bulbar onset [98]. Moreover, in this study, like for the work by Turner and colleagues [88], [18F]DPA-714 PET highlighted the involvement of an extra-motor area, namely the temporal lobe, at the early stages of the disease.

[^11^C]-PBR28 is currently one of the most promising TSPO-ligand in ALS preclinical and clinical studies, thanks to its almost 80-fold higher affinity for the receptor and good brain pharmacokinetics as compared to PK11195 [77]. Interestingly, multimodal imaging studies, combining [^11^C]-PBR28 PET with Diffusion Tensor Imaging (DTI) and/or Magnetic Resonance Spectroscopy (H-MRS), highlighted a striking correlation of increased PBR28 binding in the motor cortex, corticospinal tract and precentral gyrus with upper motor neuron burden, gray matter atrophy, and decreased subcortical fractional anisotropy [99]. However, it must be underlined that the affinity of [^11^C]-PBR28 for TSPO is significantly affected by a polymorphism in TSPO gene (*rs6971*) leading to an Ala147-Thr amino-acid substitution [100,101]. For this reason, genotyping of patients must be performed to discriminate poor binders before enrolling in [^11^C]-PBR28 PET imaging trials.

Despite extensive use of TSPO ligands in clinical and pre-clinical neuroradiological settings, the role played by TSPO+ microglia cells in the neuroinflammatory process in vivo, remains elusive. Interestingly, together with miR-155 upregulation, also TSPO and CD68 transcripts have been found upregulated in post-mortem spinal cord samples from ALS patients (see supplementary info in ref. [102]), highlighting a potential correlation between the neuroinflammation highlighted by TSPO upregulation and aberrant microglia activation (linked to miR-155 deregulation). Very recently, microglia activation was monitored in familial ALS by using [^11^C]-PK11195 and PET imaging comparing healthy subjects with asymptomatic and symptomatic SOD1 mutated carriers [45]. In this study, increased PK11195 binding was observed both in symptomatic patients as well as in asymptomatic mutant SOD1 carriers, suggesting that early neuroinflammatory responses might be engaged already during the presymptomatic phase of the disease. Notably, the regions highlighted by PK11195 binding differed among patients carrying a SOD1 mutation responsible for a severe, moderate or slow disease progression; overall this supports the prognostic value of TSPO PET-imaging. However, a detailed analysis of the genetic and functional signatures of microglia in the CNS regions highlighted by TSPO-selective PET tracers is still missing and urgently needed in order to shed light on the role and on the involvement of TSPO upregulation in the disease process.

More recently, specific increased expression of cannabinoid receptor type 2 (CB2r) and purinergic P2X7 (P2X7r) receptors have been reported in activated microglia cells in ALS rodent models and patients’ post-mortem samples. CD68 immunostaining and TSPO-ligand binding were increased in CNS areas displaying positivity for CB2r and P2X7r, supporting the association of these markers with compromised CNS districts characterized by ongoing neuroinflammatory processes and microglia cell activation [103]. This has prompted the development of radioligands targeted to CB2r and P2X7r as sensitive PET tracers alternative/complementary to TSPO-ligands, with some promising candidate compounds already validated at pre-clinical level [104]. Of the two known cannabinoid receptors, cannabinoid receptor type 1 (CB1r) is the most abundantly expressed receptor in the CNS, localized on both neurons and glial cells. CB2r is instead mainly expressed on microglia. In physiologic conditions, CB2r expression in the brain is low and present only in the cerebellum and pons, whereas it is highly upregulated under stressful conditions or during inflammatory responses; notably, *CB2r* was found overexpressed in ALS at the end stage of the disease in the spinal cord district and the brainstem [44,105,106]. CB2r is physiologically involved in regulation of long-term memory [107,108], neuronal progenitor cell proliferation [109], axon guidance [110], synaptic signaling and plasticity [111], and nociception [112,113]. Induction of CB2r expression favors microglia cell recruitment at site of damage [114,115] and aids tissue recovery [116,117] by limiting pro-inflammatory cytokines secretion, NF-κB signaling and expression of NOX-2 and Cyclooxygenase-2 (COX-2) [118,119,120]. Treatment of SOD1.G93A mice with AM1241 (a CB2r-selective agonist) resulted in delayed disease progression and extended lifespan, highlighting CB2r+ microglia cells as a promising target for therapy [121]. A similar effect on disease progression and overall survival was reported in ALS after administration of P2X7r-antagonists [108]. Here the treatment determined a downregulation of pro-inflammatory markers NADPH-oxidase and IL-1β and concomitant upregulation of cytoprotective interleukin 10 (IL-10) and Brain-derived Neurotrophic Factor (BDNF), pointing to P2X7+ microglia as a key player in the disease process [122].

[^11^C]A-836339 is the first CB2 radiotracer developed and successfully tested at preclinical level. In preclinical studies [^11^C]A-836339 was able to effectively reach the CNS and selectively bind CB2 receptor in LPS-induced mouse model of neuroinflammation and in AD mice [122]. [^11^C]A-836339 highlighted CNS regions characterized by neurodegeneration prior to symptoms onset in AD mice [123]. [^11^C]NE40, a A-836339 derivative, demonstrated promising safety profile, high and homogeneous brain uptake and rapid brain washout both in rats and primates [124], resulting in eligible for human testing [125]. Interestingly, a comparative study where PK11195 and NE40 were investigated in parallel in a mouse model of stroke highlighted that CB2r PET imaging can highlight a subset of microglia cells appearing in the early stages of neuroinflammation and endowed with potential neuroprotective function, whereas TSPO is engaged in the later stages of neurodegeneration [126]. To date, [^11^C]NE40 is the only one CB2 PET tracer tested in vivo in humans. Only two recently developed CB2r ligands, [^11^C]KD2 and [^18^F]3, have been tested in ALS, on human post mortem spinal cord samples, demonstrating high specificity for the affected CNS areas [127,128,129]. On the other hand, also P2X7r is being investigated for PET imaging of neuroinflammation, however, only few preclinical studies are available in the literature and they are limited to LPS-induced neuroinflammation mouse models [130]. Table 2 summarizes key features of the most studied PET tracers of neuroinflammation.

Altogether these observations suggest that tracking TSPO or CB2r/P2X7r could provide important readouts about ongoing neuroinflammatory processes in the pathological CNS, helping to identify the sites of microglia activation and potentially allowing to discriminate among glial cells displaying different phenotypes. Intriguingly, PET imaging studies highlighted neuroinflammation also in extra-motor CNS regions in ALS patients. Indeed, investigating differences in the gene signatures displayed by activated microglia in the affected motor regions versus the extra-motor districts could shed light on novel mechanisms involved in the pathology. In this scenario, novel markers or insights on microglia phenotypes, obtained from gene expression and single-cell/spatial transcriptomic studies, could pave the way for the discovery of additional disease-specific tracers to be exploited in the quest for non-invasive diagnostic/prognostic predictors in PET-imaging analyses.

## 6. Conclusions

Reactive microgliosis in ALS as well as in other neurodegenerative diseases has been for long considered a secondary effect of neuronal demise, i.e., a response induced in the innate immune system cells of the CNS to favor scavenging of neuronal debris. Indeed, over the past ten years several studies have underlined a central role for microglia in shaping the neuronal microenvironment towards a neurosupportive versus neurotoxic trajectory depending on the disease stage and CNS region. Understanding the molecular mechanisms driving the shift towards different phenotypes is critical for development of more efficacious strategies to manipulate the neuroinflammation. Notably, thanks to the introduction of novel advanced high-throughput molecular analyses (which include single-cell RNAseq, spatial transcriptomics and CyTOF) microglial researchers have started dissecting the complex and heterogeneous signature of microglia cells not only in the context of complex diseases such as ALS [39,45,56], but also during aging [56] and in the healthy brain both along post-natal development [51] and in adulthood [136]. This supports the hypothesis that microglia should not be considered like a homogeneously polarized cell population; rather, it displays an intrinsic spatial diversity and molecular heterogeneity which accounts for the complex role exerted in ALS pathology. Interestingly, in AD, a disease-associated microglia signature microglia (DAM) has been identified and to date researchers are starting dissecting its role in the disease process [56]. Despite a DAM-like signature has been observed also in ALS rodent models [56] and patients samples [64], several studies highlighted additional genes (listed in Table 1b) that do not belong to DAM, suggesting that ALS microglia might display a disease-specific and variable signature (influenced by the extent of neuronal demise occurring in different CNS regions variably affected by the pathology). As a consequence, given the complex and intricate network of cell-to-cell interactions and communications occurring in the CNS, it becomes urgent to investigate and dissect also the signature of astrocytes, oligodendrocytes, and other non neuronal cell types further than microglia. Indeed, the application of single-cell transcriptomics is already uncovering novel clues on the intrinsic molecular and functional heterogeneity of astrocytes and oligodendrocytes in the healthy brain [137,138,139]. It will be intriguing to explore how ALS pathology shapes the signatures of these cell types, potentially paving the way for new therapeutic target discovery.

## Figures and Tables

**Table 1:** (**a**) Gene-signature of homeostatic microglia; (**b**) gene signature of ALS microglia; (**c**) gene signature induced by LPS stimulation.

(a) Homeostatic microglia				
Gene	Species	CNS Regions Analyzed	Validation Methods	References
*Fcrls*	*Mus musculus* (C57BL6)	Brain and SC	RNA-seq, RT-qPCR, IHC, FACS	[47,48,49,50]
*P2ry12*	*M. musculus* (C57BL6)	Brain and SC	RNA-seq, RT-qPCR, IHC, FACS	[47,49,51,52,53]
*Tmem119*	*M. musculus* (C57BL6)	Brain and SC	RNA-seq, RT-qPCR	[39,47,49,50,51,53]
*Tgfbr1*	*M. musculus* (C57BL6)	Brain and SC	RNA-seq	[47,54]
*Csfr1*	*M. musculus* (C57BL6)	Brain and SC	RNA-seq	[47,54]
*Sparc*	*M. musculus* (C57BL6, DBA/2J & C57/SJL)	Brain and SC	RNA-seq, RT-qPCR	[47,55]
*Cx3cr1*	*M. musculus* (C57BL6, DBA/2J & C57/SJL)	Brain and SC	RNA-seq, RT-qPCR	[47,50,52,53,55]
*Hexb*	*M. musculus* (C57BL6)	Brain and SC	RNA-seq, RT-qPCR	[47,52]
*Olfml3*	*M. musculus* (C57BL6)	Brain and SC	RNA-seq, RT-qPCR, IHC, FACS	[39,47,49,50]
*Ltc4s*	*M. musculus* (C57BL6)	Brain and SC	RNA-seq	[47,50]
*SiglecH*	*M. musculus* (C57BL6)	Brain and SC	RNA-seq, IHC, FACS	[39,47,49,51]
*Gpr34*	*M. musculus* (C57BL6)	Brain and SC	RT-qPCR, RNA-seq	[47,49,50]
**(b) ALS microglia**				
**Gene**	**Species**	**CNS Regions Analyzed**	**Validation Methods**	**References**
*Axl* *	*M. musculus* (B6/SJL-SOD1G93A, C57BL6-SOD1G93A)	SC	Microarray, RNA-seq, scRNA-seq	[39,45,56]
*Apoe* *	*M. musculus* (B6/SJL-SOD1G93A, C57BL6-SOD1G93A)	SC	Microarray, RNA-seq, scRNA-seq	[39,45,56]
	*Homo sapiens* (ALS patients)	Lumbar SC	RNA-seq, scRNA-seq, RT-qPCR, IHC, FACS	[45]
*Spp1* *	*M. musculus* (C57BL6-SOD1G93A)	SC	RNA-seq, scRNA-seq, RT-qPCR	[39,56]
*Csf1* *	*M. musculus* (B6/SJL-SOD1G93A, C57BL6-SOD1G93A)	Brain and SC	Microarray, RNA-seq, scRNA-seq	[45,54]
*Cybb* (*Nox2*)	*M. musculus* (C57BL6-SOD1G93A)	SC	RNA-seq, RT-qPCR, IHC	[39,40]
*Igf-1*	*M. musculus* (C57BL6-SOD1G93A)	SC	RNA-seq, RT-qPCR	[39]
*Grn*	*M. musculus* (C57BL6-SOD1G93A)	SC	RNA-seq, RT-qPCR	[39]
*Optn*	*M. musculus* (C57BL6-SOD1G93A)	SC	RNA-seq, RT-qPCR	[39]
*Mmp-12*	*M. musculus* (C57BL6-SOD1G93A)	SC	RNA-seq, Microarray	[39,57]
Tyrobp * (Dap-12)	*M. musculus* (C57BL6-SOD1G93A)	SC	RNA-seq, scRNA-seq, RT-qPCR, ST	[39,56,58]
	*H. sapiens* (ALS patient)	Cervical and lumbar SC	ST	[58]
Trem2 *	*M. musculus* (C57BL6-SOD1G93A)	SC	RNA-seq, scRNA-seq, ST	[56,58]
	*H. sapiens* (ALS patient)	SC	ST	[58]
Lpl *	*M. musculus* (C57BL6-SOD1G93A)	SC	RNA-seq, scRNA-seq, ST	[56,58]
	*H. sapiens* (ALS patient)	Cervical and lumbar SC	ST	[58]
B2m *	*M. musculus* (C57BL6-SOD1G93A)	SC	RNA-seq, scRNA-seq, ST	[56,58]
	*H. sapiens* (ALS patient)	Cervical and lumbar SC	ST	[58]
Ctsl *	*M. musculus* (C57BL6-SOD1G93A)	SC	RNA-seq, scRNA-seq	[39,56]
Itgax *	*M. musculus* (C57BL6-SOD1G93A)	Brain & SC	RNA-seq, scRNA-seq	[54,56]
Clec7a *	*M. musculus* (C57BL6-SOD1G93A)	SC	RNA-seq, scRNA-seq	[39,54,56]
**(c) LPS-stimulated microglia**				
**Gene**	**Species**	**CNS Regions Analyzed**	**Validation Methods**	**References**
*Stat3*	*M. musculus* (C57BL6, DBA/2J and C57/SJL)	Brain and SC	RNA-seq	[39,55]
*Socs3*	*M. musculus* (DBA/2J and C57/SJL)	Brain	RNA-seq	[49,55,59]
*Map3k8*	*M. musculus* (DBA/2 J and C57/SJL)	Brain	RNA-seq	[55]
*Ccl2*	*M. musculus* (C57BL6)	Brain	RNA-seq	[49]
*Gpr84*	M. musculus (C57BL6)	Brain	RT-qPCR	[49,60,61]

* = disease-associated microglia (DAM)-like gene signature; scRNA-seq = single-cell RNA-seq; ST = spatial transcriptomics; IHC = immunohistochemistry; FACS = fluorescence activated cell sorting; SC = spinal cord.

**Table 2:** Pet tracers of neuroinflammation.

Tracer and Radioisotope	Target	Tested in	Notes	References
[11C]-(R)-PK11195	TSPO	human ALS patients and healthy controls	signals are not influenced by patient’s TSPO genotype	[103,131]
		rat model of cerebral ischemia (Wistar rats)	poor specificity	
[18F]DPA-714	TSPO	ALS model (SOD1G93A mouse)	signals correlate to increased TSPO expression and compromised brain regions	[87,131]
		rat model of cerebral ischemia (Wistar rats)	higher affinity and better signal-to-noise ratio than PK11195	
[11C]-PBR28	TSPO	human ALS, PLS patients and healthy controls	signals correlate to glial activation and inflammation	[102,132]
		human healthy subjects	binding is affected by TSPO polymorphism	
[11C]A-836339	CB2	neuroinflammation-induced/AD models (CD-1 and APPswe/PS1ΔE9 mouse)	first CB2 radiotracer tested	[123,124]
		AD model (APPswe/PS1ΔE9 mouse)	detection of neuroinflammation very early in the pathology	
[11C]NE40	CB2	AD, PD patients and healthy controls	no differences between disease and control cases	[127,128,133]
		ischemic stroke model (Sprague–Dawley rat)	signal in peri-infarct area, concomitant to CB2 up-regulation	
		senescence-accelerated model (SAMP10 mouse)	detection of early signs of neuroinflammation in cortex	
[11C]KD2	CB2	ALS patients	selective binding in post-mortem ALS spinal cord specimens	[129,134]
		neuroinflammation-induced model (CD-1 mouse)	limited target specificity and excessive lipophilicity	
[11C]RS-016	CB2	neuroinflammation-induced model (CD-1 mouse)	high blood stability and CB2 specificity	[134]
		ALS patients	selective binding in post-mortem ALS spinal cord specimens	
[18F]29	CB2	neuroinflammation-induced model (CD-1 mouse)	CB2 specific tracing but very rapid metabolism	[105]
[18F]3	CB2	Wistar rats	rapid washout from brain tissue	[130]
		ALS patients and healthy controls	selective binding in post-mortem ALS spinal cord specimens	
[11C]GSK1482160	P2X_7_	neuroinflammation-induced model (C57BL6)	increased signals in CNS sites with prominent neuroinflammation	[135]

## Abbreviations

AD, Alzheimer’s Disease; ALS, Amyotrophic Lateral Sclerosis; ApoE, Apolipoprotein E; B2m, Beta-2-Microglobulin; BDNF, Brain-derived Neurotrophic Factor; CB1r, Cannabinoid Receptor type 1; CB2r, Cannabinoid Receptor type 2; Clec7a, C-type lectin domain family 7 member A; CNS, Central Nervous System; COX-2, Cyclooxygenase-2; CSF, Cerebrospinal Fluid; Cx3cr1, C-X3-C Motif Chemokine Receptor 1; CyTOF, flow cytometry by time-of-flight mass spectrometry; DAM, Disease-Associated Microglia; DAP12/Tyrobp, Transmembrane Immune Signaling Adaptor; DTI, Diffusion Tensor Imaging; ER, Endoplasmic Reticulum; FACS, flow cytometry; fALS, Familiar ALS; FTD, Frontotemporal Dementia; GWAS, Genome Wide Association Studies; H-MRS, Proton Magnetic Resonance Spectroscopy; IHC, immunohistochemistry; IKK, Inhibitory-κB Kinase; Itgax, Integrin alpha X; LMN, Lower Motor Neurons; Lpl, Lipoprotein lipase; LPS, Lipopolysaccharide; MAMs, Mitochondria-Associated ER Membranes; Matr3, Matrin 3; MMP-12, Metallopeptidase 12; MNs, Motor Neurons; MPTP, Membrane Permeability Transition Pore; MRI, Magnetic Resonance Imaging; NADPH, Nicotinamide Adenine Dinucleotide Phosphate reduced; NF-κB, Nuclear Factor Kappa-light-chain-enhancer of Activated B Cells; NOX2, NADPH-Oxidase 2; OPTN, Optineurin; PBR, Peripheral-type benzodiazepine receptor; PD, Parkinson’s Disease; PET, Position Emission Tomography; Pfn1, Profilin 1; ROS, Reactive Oxygen Species; sALS, Sporadic ALS; SC, Spinal Cord; TREM2, Triggering Receptor Expressed on Myeloid Cells 2; TSPO, Translocator Protein; UMN, Upper Motor Neurons; VDAC, Voltage-Dependent Anion Channel; VEGF, Vascular Endothelial Growth Factor; WNT, Wingless/Integrated.
